# Onco-mNGS facilitates rapid and precise identification of the etiology of fever of unknown origin: a single-centre prospective study in North China

**DOI:** 10.1186/s12879-024-10383-3

**Published:** 2024-12-28

**Authors:** Bingbing Liu, Tengfei Yu, Ruotong Ren, Na Wu, Nanshu Xing, Jingya Wang, Wenjie Wu, Xuefang Cao, Jingping Zhang

**Affiliations:** 1 Department of Infectious Diseases, The First Hospital of China Medical University, Shenyang, 110001 China; 2 Micro-Health Biotechnology Co., Ltd, Hefei, 230001 China; 3 Institute of Innovative Applications, MatriDx Biotechnology Co., Ltd, Hangzhou, 311112 China; 4 Faculty of Life Science and Technology, Kunming University of Science and Technology, Kunming, 650500 People’s Republic of China; 5 Foshan Branch, Institute of Biophysics, Chinese Academy of Sciences, Beijing, 100101 China

**Keywords:** Fever of unknown origin, Onco-mNGS, Etiology, Diagnostic criteria

## Abstract

**Objectives:**

Delayed diagnosis of patients with Fever of Unknown Origin has long been a daunting clinical challenge. Onco-mNGS, which can accurately diagnose infectious agents and identify suspected tumor signatures by analyzing host chromosome copy number changes, has been widely used to assist identifying complex etiologies. However, the application of Onco-mNGS to improve FUO etiological screening has never been studied before.

**Methods:**

In this single-centre prospective study, we included 65 patients with classic FUO, who were randomly divided into control group (sample cultivation) and mNGS group (cultivation + Onco-mNGS). We analyzed the infectious agents and symbiotic microbiological, tumor and clinical data of both groups.

**Results:**

Infection-related pathogenic detection efficiency rose from 25% (control group) to 48.48% (experimental group). Seven patients with chromosome copy number changes had later been confirmed tumors, indicating a 100% of clinical concordance rate of Onco-mNGS for tumors. In addition, the time frame for diagnosing or ruling out infection/tumor with Onco-mNGS had greatly reduced to approximately 2 days, which was 7.34 days earlier than that in the control group.

**Conclusions:**

Onco-mNGS is an ideal rapid diagnostic aid to assist improving the early diagnostic efficiency of FUO-associated diseases.

**Supplementary Information:**

The online version contains supplementary material available at 10.1186/s12879-024-10383-3.

## Introduction

Fever of Unknown Origin (FUO) is a clinical manifestation characterized by fever lasting over 3 weeks, oral temperature exceeding 38.3℃ on 3 occasions, or temperature fluctuating by over 1.2℃ within 1 day without confirmed diagnostic results after a week of thorough examinations in an outpatient or inpatient setting [1]. It is commonly observed in infectious diseases, neoplasm, immune and inflammatory processes [2]. Fever can be divided into four categories: the typical fever to be investigated and the fever to be investigated in the special population, the fever to be investigated in the special population includes the fever to be investigated in hospitalized patients, the fever to be investigated in patients with agranulocytosis and the fever to be investigated in HIV infected persons. They are different in terms of disease spectrum and diagnosis and treatment process, and the definition described in the article is the classic definition of FUO. Because of the etiological complexity, clinical diagnosis of FUO has long been a challenge for physicians. Although standardized diagnostic approach that being proposed by Ghady Haidar and Nina Singh [2] had improved the diagnostic efficiency of FUO to some extent, there were still 8.35%—32% of FUO patients ended up with undiagnosed illness [3–7].

Among the documented etiologies that cause FUO, infections and neoplasms were the two leading causations [8, 9]. Although numerous methods had been routinely used for infection- and neoplasm- diagnoses, disadvantages of these conventional approaches in FUO diagnoses irreversible started to emerge over time in clinical practices, especially for those caused by infections and neoplasms. For example, the diagnosis of infection commonly relied on sample cultivation, supplemented by PCR and immunological methods. However, apart from problems such as low pathogen detection rate and long diagnostic time that frequently occurred for these methods, they were unable to identify rare or unknown pathogens which might result in infection-mediated FUO. Moreover, clinical screening of malignancies generally involved abdominal ultrasound, posterior chest radiography, chest/abdomen/pelvis computed tomography (CT) scans, or puncture pathology biopsy [2], which were not only time-consuming but also with low-positive rates.

With advances in technology, metagenomic next-generation sequencing (mNGS) that allows for unbiased identification of pathogenic microorganisms had been increasingly used for early diagnoses of infection-induced FUO [10–12]. However, conventional mNGS methods were applicable only for infection-associated diseases, but not for those caused by tumors. To fill in this technical gap, a new procedure named Oncology mNGS (Onco-mNGS) technology recently emerged which allowed precise identifications of both pathogens and human chromosome copy number variations (CNVs) in clinical samples with a one-step approach [13–15]. In our prior studies, Onco-mNGS had a sensitivity of 93.04% and a specificity of 60.00% for pathogenic identification [16], and a clinical compliance rate of 100% and a positive detection rate of 83.7% for aberrant tumour-associated CNV signals in body fluid samples [14].

In this study, we compared the diagnostic efficiency of between Onco-mNGS and conventional diagnostic methods in 65 classic FUO patients. Depending on the outstanding diagnostic efficacy of FUO in terms of detection sensitivity, accuracy, and time frame, we confirmed the avdantages of Onco-mNGS in complementing clinical diagnosis of FUO.

## Materials and methods

### Patients and study design

This is a single-center prospective study which involves 65 patients with classic FUO at the First Affiliated Hospital of China Medical University between the 1st of May and 30th of September in 2021. The patients or their families provided consent for the samples before enrollment and signed the informed consent form for blood collection and study.

The enrolled patients were randomly divided into experimental and control groups. They underwent routine biochemical tests, while samples were collected according to the clinical needs and the samples were simultaneously subjected to Onco-mNGS analysis and conventional microbiological cultures. The microbiological detection method was a combination of Onco-mNGS and culture in the experimental group and culture only in the control group.

### DNA extraction and mNGS

Based on the mNGS Automatic Library Preparation System (Cat. MAR002, MatriDx Biotechnology Co., Ltd. Hangzhou, China), all clinical samples were subjected to DNA extraction, library preparation and mNGS (50-bp single-end reads; Illumina). The abbreviated steps were as follows: 1) DNA extraction and library preparation were performed on an NGS automated library preparation system. Relevant reagents include: Nucleic Acid Extraction Kit (Cat. MD013, MatriDx Biotechnology Co., Ltd. Hangzhou, China), Cell-free DNA Library Preparation Kit (blood samples) (Cat. MD007, MatriDx Biotechnology Co., Ltd., Hangzhou, China) and Total DNA Library Preparation Kit (other sample types) (Cat. MD001T, MatriDx Biotechnology Co., Ltd., China); 2) Libraries were pooled and then sequenced on an Illumina NextSeq500 system using a 75-cycle sequencing kit.

## CNV and Pathogen Detection With mNGS Data Simultaneously

We used Onco-mNGS to look for both pathogen and tumor clues through mNGS data simultaneously. The method is as follows. Sequenced reads were first compared to the human reference genome (hg19) from the NCBI database, and only uniquely positioned reads were selected for subsequent analysis. The reference genome was partitioned into contiguous windows of fixed length, and read depths were calculated for each window and then normalised to the total number of reads per sample. The copy number ratio for each window was obtained by dividing the normalised read depth by the average read depth in the reference dataset. The copy number is then transformed to log2 and adjacent open frames with similar ratios are combined into segments annotated with chromosome position and average ratio. The copy number of each segment was calculated based on the mean ratio and normal copy number of the corresponding chromosome and then compared to a preset threshold to validate CNV.

Clean reads obtained after raw data demultiplexing, adapter trimming and human reads removing were subjected to microbial identification based on a reference database containing over 20,000 microorganisms. All species detected in clinical samples using mNGS are first filtered with all microorganisms detected in the parallel no template control (NTC) (background microorganisms) with a ratio of unique reads per million (RPM) above 10, and the RPM ratio = RPM_sample_/RPM_NTC_ or RPM ratio = RPM_sample_ if the organism was not detected in the parallel NTC. All the species authentically present in clinical samples are defined as microbiota. Substantially, all species of microbiota were looked up in PubMed (https://pubmed.ncbi.nlm.nih.gov/) to determine whether the organisms cause infection and the positive pathogenic microorganisms were defined as pathogens. Filtration is considered for human colonizing microorganisms, rather than pathogenic bacteria, such as oral anaerobic bacteria, enterobacter cloaca, human herpes virus, etc. Finally, potential pathogens were selected from the results of previous analyses according to the clinical phenotype and these data were reviewed by senior clinicians. For the diagnosis of infectious diseases, combined with clinical manifestations, physical examination, related laboratory, imaging examination and mNGS results, to obtain the final diagnosis. However, staphylococcus hominis, staphylococcus epidermidis, and oral anaerobic bacteria are considered to be human colonization bacteria, rather than responsible pathogens, and should be ignored. For the diagnosis of non-infectious diseases, the possibility of infectious fever should be fully considered clinically, combined with antibody, pathological or cytological testing, and specialists (oncology, rheumatology, etc.) should be consulted to assist in the final diagnosis.

### Diagnosis of Infection and Tumor

The results of the etiological screening of the patients were evaluated by a panel of clinical experts, including three experienced physicians and a clinical microbiologists. mNGS results were interpreted according to the standard data processing workflow of MatriDx Biotechnology Co., Ltd. Infections are diagnosed on the basis of microbiological tests, mNGS results and clinical review results. Tumors are judged on the basis of Onco-mNGS results in addition to histopathology, cytological examination, microscopic examination and other validation tests. Clinical impact was arbitrated by all authors according to the project's pre-defined clinical impact rubric after review and discussion of each case by the treatment team.

### Statistical analysis

Statistical analysis was performed by SPSS 26.0. Alpha diversity was estimated on basis of the expression profile of each sample according to the Shanon, Simpson, simpson, Chao 1 index. Beta diversity was estimated by the Bray–Curtis dissimilarity between samples. A *p*-value < 0.05 was considered as statistically significant.

## Results

### Basic information of the enrolled patients

**Fig. 1:**
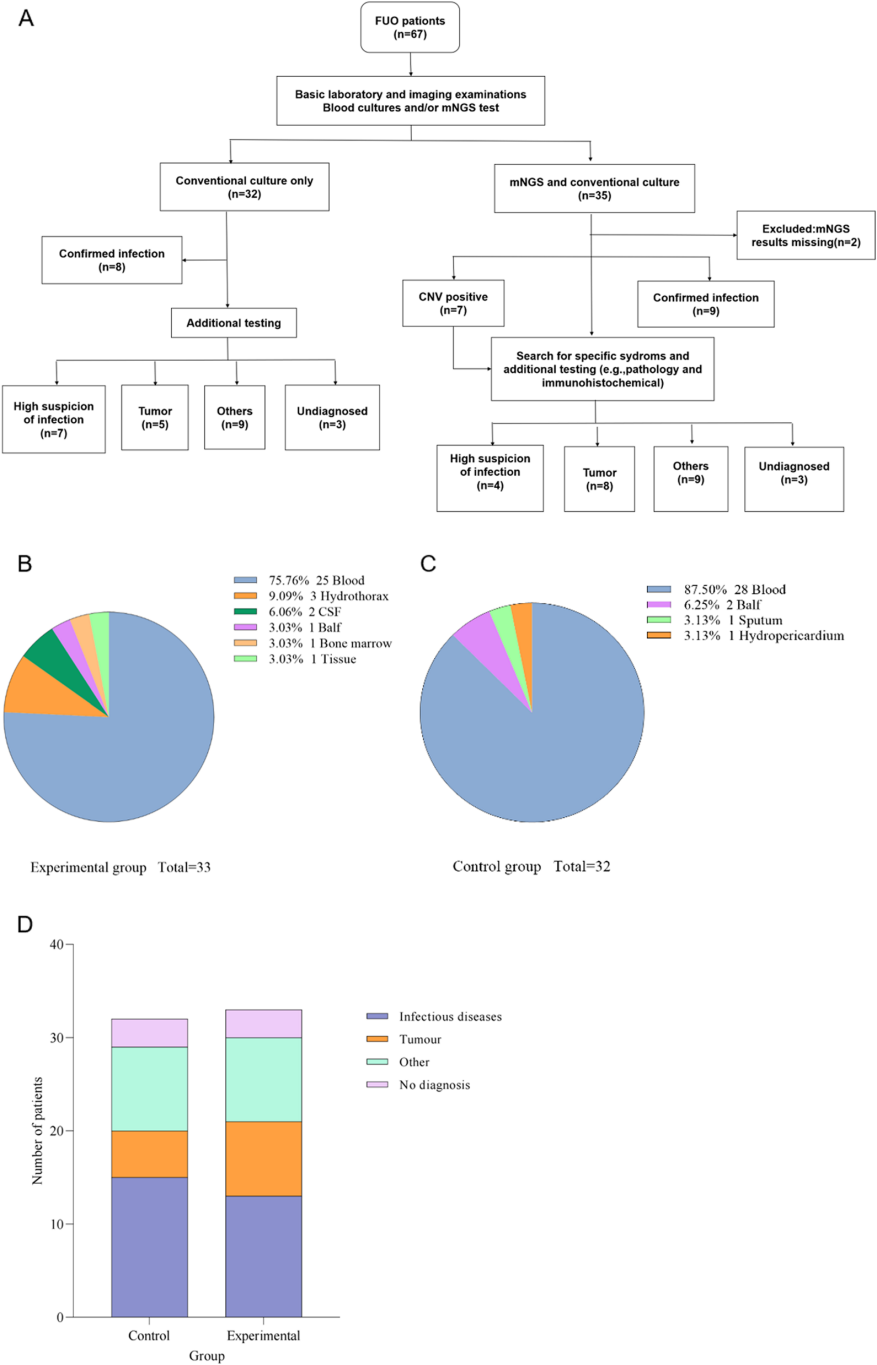
Flowchart of Research, Clinical grouping and patient distribution. **A** Flowchart of this research, **B** Sample types in Control group, **C** Sample types in Experimental group, **D** Patient distribution with four different etiologies in two groups

Two of the 67 patients initially enrolled in this study were eliminated due to low validated mNGS data, and 65 were eventually enrolled, including 32 (49.23%) males and 33 (50.77%) females. These patients were randomly assigned to experimental (n = 33) and control (n = 32) groups, then undergoing the corresponding treatment protocols (Fig. 1A). Table 1 and Supplementary Material Table S-1 listed the baseline and initial lab results. A total of 3 alveolar lavage, 3 pleural fluid, 2 cerebrospinal fluid, 1 sputum, 1 pericardial fluid, 1 bone marrow, 1 tissue, and 53 blood samples were included in this study (Fig. 1B, 1C and Supplementary Material Fig. S-1).

**Table 1:** Demographic and etiological information of patients

	Experimental group Control group	Experimental group Control group
Number of people	33	32
Age, mean	58.81 (26, 85)	51.13 (18, 81)
Sex, Female (n%)	16 (48.48%)	17 (53.13%)
Disease types		
Infectious diseases	13	15
Tumour	9	5
Other	8	9
FUO	3	3

Other, Autoimmune disease, inflammatory disease and other non-infectious non-neoplastic diseases; FUO, fever of unknown origin; Data were presented as n (%) or means (range).

Of the enrolled 65 patients, 59 (90.77%) were diagnosed after routine pathogenesis, imaging, Onco-mNGS testing or clinical experience of physicians, while 6 (9.23%) still had an unknown cause of fever after repeated testings. The confirmed cases included 28 cases (43.07%) of infections, 13 (20.00%) of tumors and 18 (27.69%) of other diseases (Fig. 1D).

### mNGS improves pathogen detection rate

**Fig. 2:**
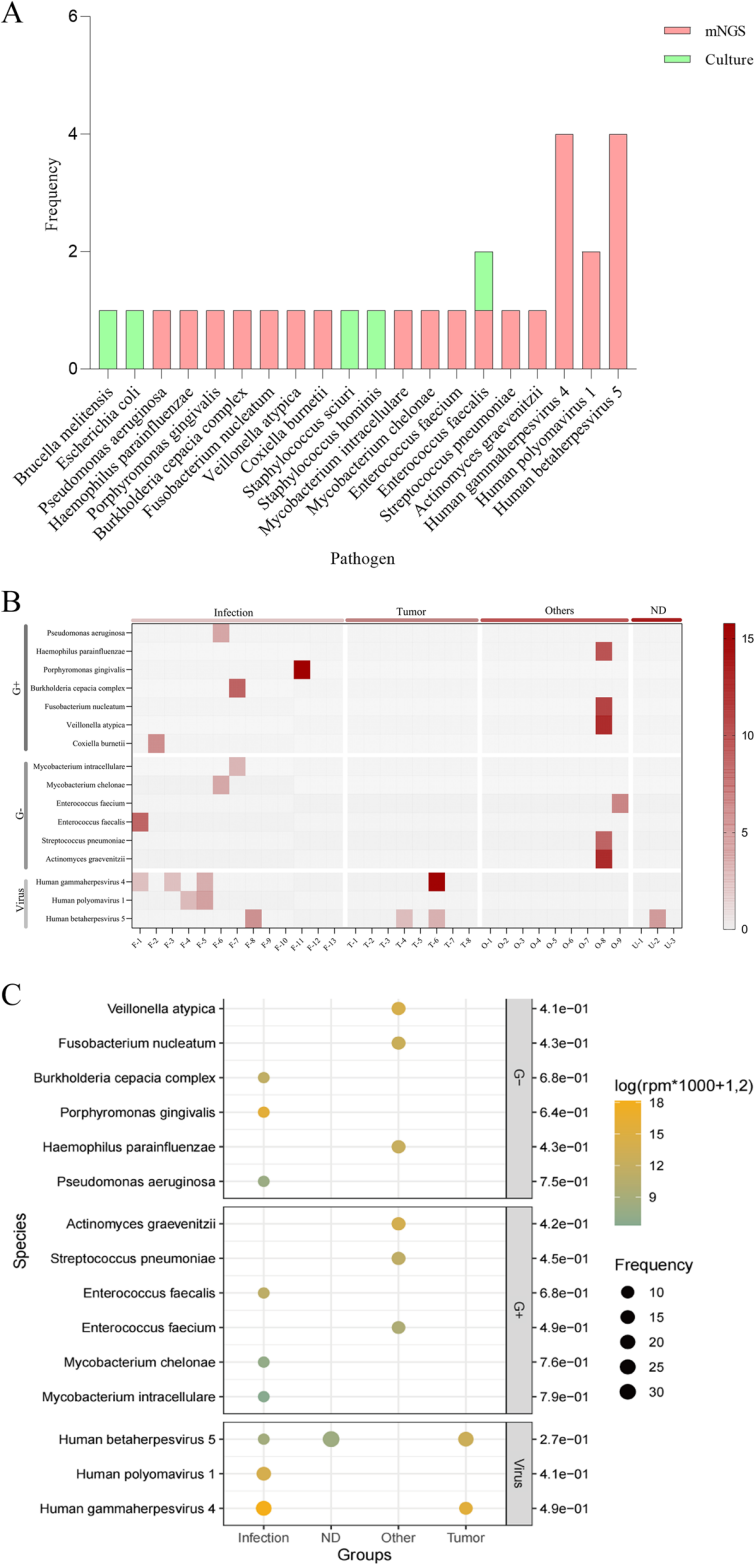
Comparison of the efficacy of Onco-mNGS and conventional culture methods for pathogenicity detection in the experimental group. **A** Analysis of responsible pathogens detected by Onco-mNGS and conventional culture methods, **B** The distribution pattern of bacteria, fungi and virus identified by Onco-mNGS in patients with different etiology, **C** Difference analysis of the abundance and frequency in three different types of responsible pathogens (bacteria, fungi, and viruses) identified by Onco-mNGS. Infection, *n* = 8; Tumor, *n* = 9; Other, *n* = 4; ND, *n* = 3. *p* < 0.05, *, *p* < 0.01, **

Based on the Onco-mNGS results, 20 pathogens were found to be causative in the experimental group (48.48%, 16/33), while 6 were found in the control group (25%, 8/32). The predominant pathogens detected in both groups were bacteria and viruses, with the frequencies of detection being 13 in the experimental group and 10 in the control group for bacteria, and 7 in the experimental group and 1 in the control group for viruses, respectively. Of the 20 responsible pathogens detected in the experimental group, four were solely obtained by sample cultivation and 15 were detected by Onco-mNGS, while *Enterococcus faecalis* had been detected by both methods (Fig. 2A). These pathogens were originated from 16 cases, 14 of which were detected by Onco-mNGS alone. These cases contained 13 single bacterial or viral infections, and one mixed infection. We also analysed the pathogen detection rates for cases of different etiology in experimental group: the pathogen detection rate for infectious FUO was 100% (13/13), which was significantly higher than those caused by tumour (50%, 4/8) and other causes like non-infectious non-tumour inflammation (55.56%, 5/9) (Fig. 2B, 2C).

### The empirical use of antibiotics had less effect on the pathogen detection results of Onco-mNGS than culture

To explore the impact of empirical antibiotic use on the test effectiveness of both methods, a statistical analysis about the empirical use of antibiotics and pathogen detection has been performed. Biodiversity is often measured and analyzed using two key components, alpha diversity and beta diversity. Alpha diversity refers to the diversity within a specific habitat or community. Beta Diversity, on the other hand, focuses on the differences in species composition between different habitats or communities.

**Fig. 3:**
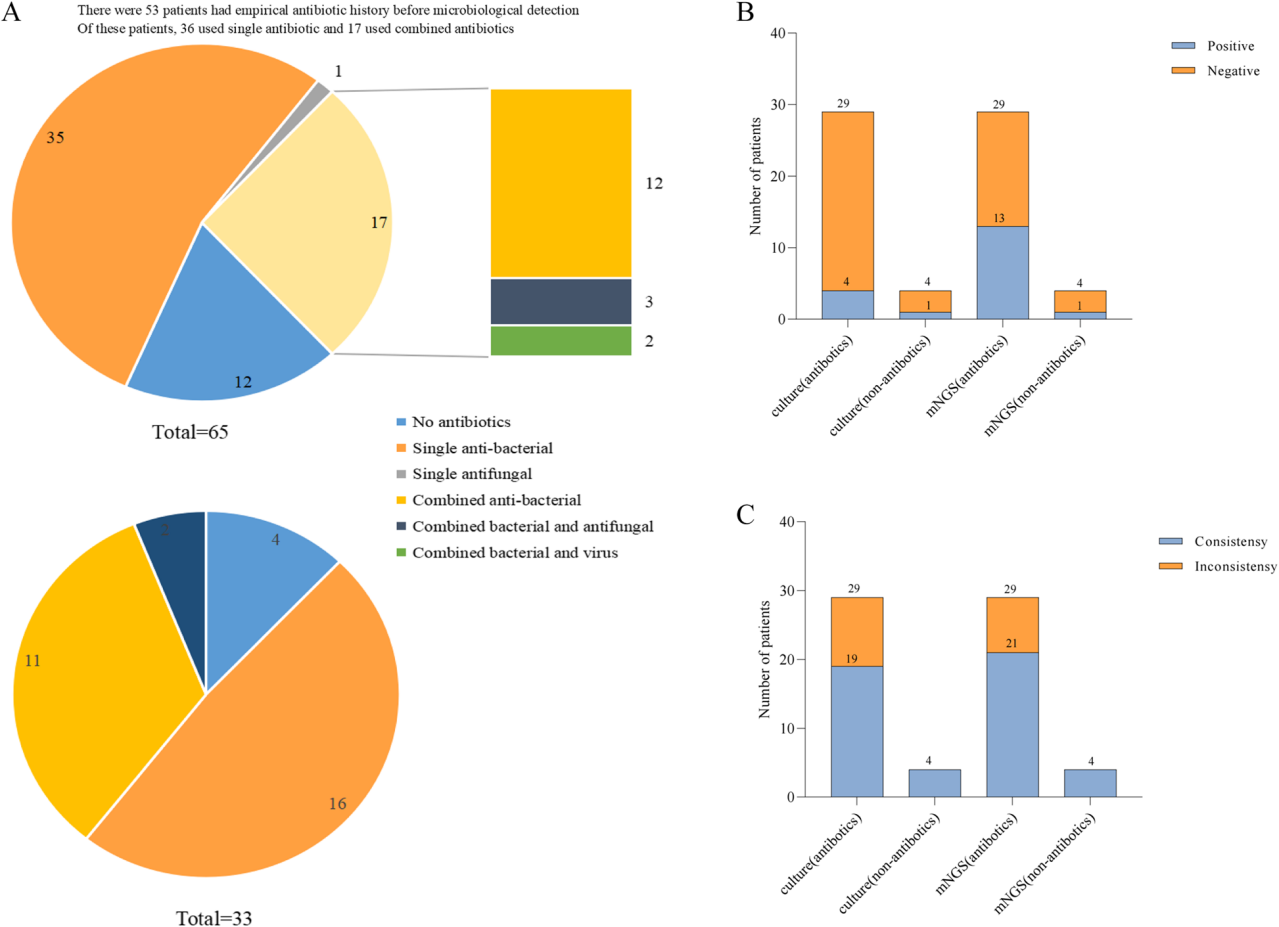
Analysis of empirical antibiotic use in experimental group. **A** Most patients in both experimental group and control group used antibiotics empirically, **B** Among the empirical anti-infective cases, 4 cases were positive from traditional culture, and 13 cases were positive from Onco-mNGS, **C** Analysis of consistency between culture and Onco-mNGS detection results and clinical diagnosis in empirical anti-infective cases

There were 53 patients had empirical antibiotic history before microbiological detection. Of these patients, 36 used single antibiotic and 17 used combined antibiotics (Fig. 3A and Supplementary Material Table S-2). Among the 29 patients in the experimental group who used antibiotics empirically, pathogens were detected in 13 cases (44.83%) by Onco-mNGS but only 4 (13.79%) by culture; among the 4 patients who did not use antibiotics, two methods both has detected 1 positive case (25%) (Fig. 3B).

After analyzing the clinical concordance between these two methods, the clinical committee found that Onco-mNGS had a clinical concordance rate of 72.4% (21/29) in empirical antibiotic used cases of the experimental group, compared to 65.5% (19/29) for culture. Both methods had 100% clinical concordance (4/4, 4/4) for patients not on antibiotics (Fig. 3C).

### Onco-mNGS improves the efficiency of tumour diagnosis

**Fig. 4:**
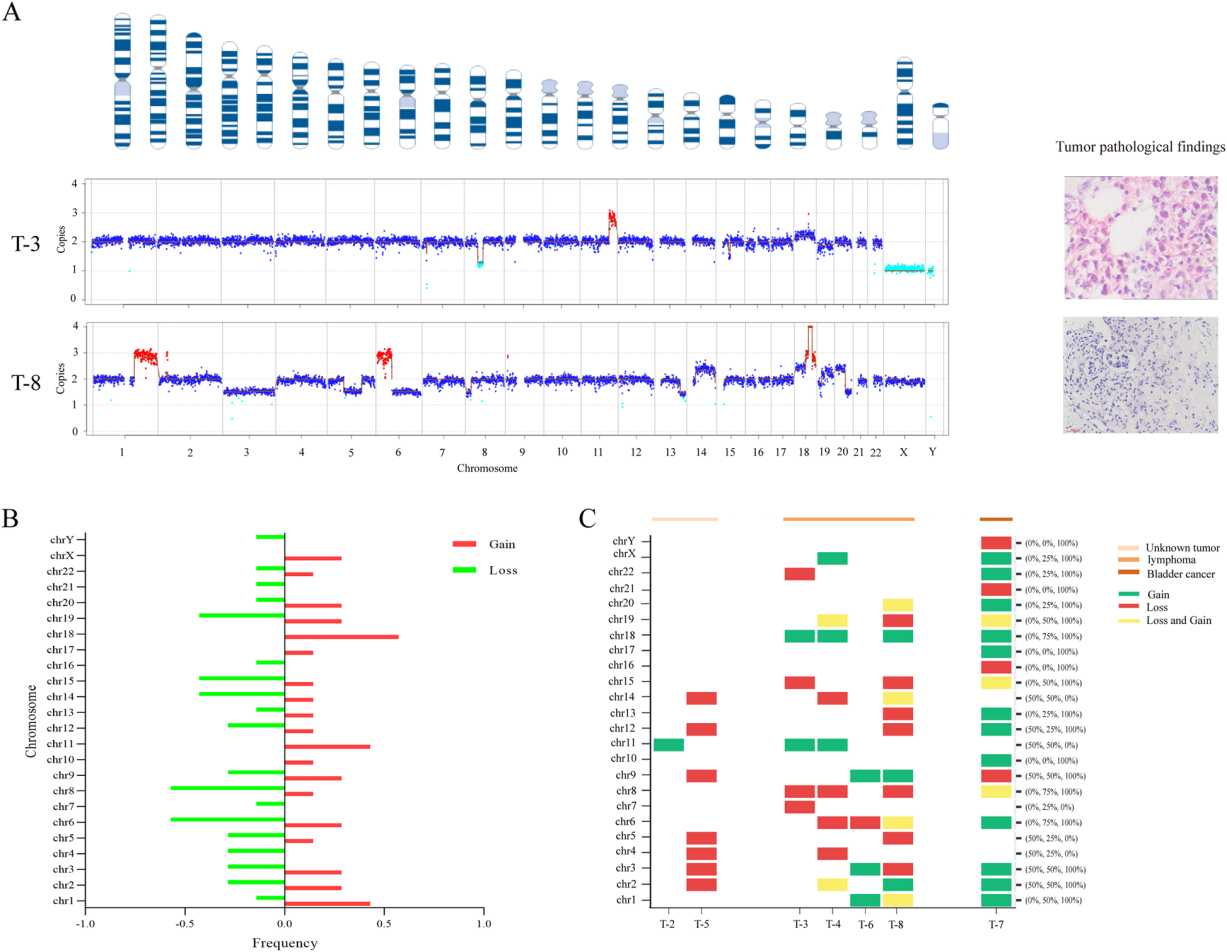
CNV analysis in patients with tumor etiology of FUO. **A** Abnormal CNV signals in tumor patients and their pathological findings: T-3 was bone biopsy, T-8 was bone marrow biopsy, **B** Chromosome variation types of 7 CNV positive patients, **C** The chromosomal variation types of different cancer species showed the solid tumor has more variation sites

CNV analysis was performed on 33 samples from the experimental group to test the tumor screening capacity of Onco-mNGS. On average, about 21 million homo reads (ranging from 8.8 to 89 million) were obtained from each sample after excluding microbial sequences. These sequenced fragments were spliced and analysed against the normal human genome sequence (hg19), and the CNV abnormalities were determined by the correspondence of chromosomal and CNV wave maps (Fig. 4A). The experimental group has seven aberrant CNV patients. Two of the patients (T-4, T-7) had previously been diagnosed as tumor patients, and one (T-5) had multiple lymph nodes in the abdominal cavity with abnormal CNV results, was considered a neoplasm. The rest of 4 patients (T-2, T-3, T-6, T-8) confirmed tumors through immunohistochemical (Supplementary Material Table S-3) or pathological staining (Fig. 4A and supplementary material Fig. S-3) results. Further analysis of CNV variant sites showed that bladder cancer patients had 79.17% (19/24) variant sites, lymphoma patients had 36.46% (8.75/24) chromosomal mutations on average(Fig. 4B, 4C).

The results above showed that Onco-mNGS has 100% of specificity and 87.5% of sensitivity for cancer screening (Supplementary Material Fig. S-4). Notably, lymphoma was diagnosed in four of these seven patients (57.14%). One patient who was negative for CNV by Onco-mNGS but had a clinically confirmed tumour was confirmed as pleural mesothelioma by CT and pleural fluid cytology.

### Onco-mNGS significantly reduces the diagnostic time

We compared the time needed for diagnosing or ruling out infection/tumor of the two groups. The average time period for experimental group (n = 30) clarify or ruling out infection/tumor was 7.3 days shorter than that of the control group (n = 29) (Supplementary Material Fig. S-5A).

In addition, we investigated the implications of Onco-mNGS for the clinical management of FUO patients. 17 of 33 experimental group patients stopped application of antibiotics after the causative agents being identified or excluded and 9 patients were transferred to other departments for further treatment. The clinical committee concluded that the Onco-mNGS results had positive implications for the diagnosis and clinical management of these 26 patients (Supplementary Material Fig. S-5B). The remaining 7 patients had a final diagnosis of inflammatory diseases or early neoplastic stage confirmed by clinical combination of antigen/antibody testing, pathology, ultrasound or CT.

## Discussion

More than 200 etiologies have been documented to contribute to FUO [5, 6, 17]. The standardized diagnostic procedure proposed by Ghady Haidar et al*.* [2]combined understanding the natural history, routine biochemical tests, and other imaging techniques to diagnose FUO. Unfortunately, this strategy is basically inaccurate, low-efficient and largely dependent on the external environment, experiences of clinicians, and characteristics of patients. Therefore, increasing clinical research has focused on improving the efficiency and accuracy of FUO etiological diagnosis.

Infection and tumor are the main causes of FUO, and William F. Wright et al*.* [9] observed in a large literature research that 37% of FUO patients had infectious illnesses. Current clinical diagnosis of infection is mostly based on microbiology laboratory culture results, which relies heavily on the physician's clinical experience, the standardization of the laboratory testing process, and the patient's epidemiological information. This method has low controllability and is unfavorable for detecting rare and emerging pathogens. In recent years, the methods of testing for FUO pathology have been continuously optimized. For example, Wanru Guo et al. [18] attempted to use complete exome sequencing technology to address the problem of FUO tumour screening. Kim-Heang Ly [19], Friedrich Weitzer [20], applied F-18 FDG PET/CT to the etiological screening of inflammatory FUO patients and compared PET/CT with thoracoabdominal-pelvic CT (CAP-CT) in terms of diagnostic direction, contribution and time. They showed that PET/CT screened FUO patients faster and more efficiently than CAP-CT and proposed using F-18 FDG PET/CT to help diagnose early. However, these approaches only screen for one etiology of FUO. Thus, a non-invasive test that can detect both pathogens and tumors would greatly improve the efficiency of FUO aetiology screen.

As an emerging molecular diagnostic technique, mNGS is increasingly used to screen pathogens in patients with suspected infections. This approach detects pathogenic gene sequences, hence it is not useful for diagnosing non-infectious disorders yet. To fill this technical gap, the new technology Onco-mNGS is being employed in clinic from 2022. This technique superimposes chromosomal CNV analysis on top of the mNGS technology, allowing simultaneous screening for infections and tumours [15]. Our study examined the use of Onco-mNGS in FUO etiological screening and found the following.

Firstly, Onco-mNGS is useful for clinical aetiological screening. In this study, we found that the experimental group had significantly higher detection efficiency than the control group in terms of pathogen type (20 vs. 6), number (23 vs. 8) and detection rate (48.48% vs. 25%). Moreover, Onco-mNGS is useful in detecting clinically emerging, rare and uncommon pathogens. This study found 16 responsible pathogens via Onco-mNGS, including *Coxiella burneti*, *Mycobacterium intracellulare*, *Mycobacterium torulare*, and viruses, which are harder to culture. *C. burneti* is an internal parasitic bacterium that eludes clinical microbiological culture procedures like Petri dishes [21], while microorganisms of the genus *Mycobacterium* and viruses are often more difficult to achieve in clinical culture due to the harsh culture conditions or long culture cycles. Most of the 20 pathogens identified as cause of FUO in experimental group are not generally associated with FUO. Most (about 60%) are non-infectious fevers, and one of the main purposes of Onco-mNGS testing is to help the clinical rapid exclusion of infections to reduce the use of unnecessary antibiotics. The pathogens detected by Onco-mNGS are basically consistent with traditional culture, but have obvious advantages in timeliness. Some special pathogens, such as Coxiella, are difficult to detect by traditional culture methods, and Onco-mNGS has absolute advantages in searching for such pathogens.

In addition to identify the responsible pathogens, Onco-mNGS is also able to output human microecological data simultaneously. In this study, we analyzed 25 blood samples from the experimental group and found that patients with infection-associated FUO had higher abundance of blood microecology than those with other etiologies and that the diversity of microecological populations were significantly different from the tumor group. High abundance of human microecology has been progressively shown to be strongly associated with the development of infections, tumors and other diseases. Kypros Dereschuk et al*.* found [22] the abundance of six microorganisms was associated with the severity of COVID-19, while the abundance of *Bacillus subtilis* had a positive correlation with the improvement of COVID-19.

Onco-mNGS could also screen abnormal CNV signals in human genome, which allows for effective early warning of tumors. The current gold standard for tumor diagnosis is pathological biopsy, however, this invasive technique required clinical confirmation of the tumor site, resulting its limited application in clinical practices. Onco-mNGS, on the other hand, is a simple and highly accurate method of screening for abnormal CNV signals using only a blood test, and can be used as a broad-spectrum early screening aid for tumors. The seven patients described in this study underwent abnormal blood CNV tests, all but two of whom had previously diagnosed tumors, and five were first diagnosed by clinical tumor screening after Onco-mNGS early warning. It was also noticeable that, a patient with a normal CNV signal but a clinically confirmed malignant pleural mesothelioma. The missed detection by Onco-mNGS in this patient might be associated with the lower mutant gene load in the blood as seen in other researches. For instance, Raphael Bueno et al*.* [23] sequenced 216 malignant pleural mesothelioma patients and found that the somatic point mutation rate of protein changes was lower than in other solid malignancies. In another cohort of 74 malignant pleural mesotheliomas, whole-exome sequencing confirmed an overall incidence of < 2 bases/M for non-synonymous mutations in all samples except for one sample with a mutation of < 8 bases/M [24]. It indicated that Onco-mNGS for CNV signalling screening is best for samples with high abnormal cell content, such as haematological tumors and tumor systemic metastases, but its sensitivity is low for early tumor or tumor with low somatic point mutation rates. Meanwhile, multipoint detection should improve the detection sensitivity.

Secondly, Onco-mNGS offers advantages in adapting clinical antibiotic regimens for FUO. In our study, up to 81.5% of febrile patients were empirically treated with antibiotics before diagnosis, some even with carbapenem or a combination of broad-spectrum antibiotics, but generally with poor outcomes. Although empirical antibiotic therapy is indicated in critical FUO patients with no definite cause but a strong suspicion of infection, many doctors are urging caution. William F. Wright [1] et al. suggested that the empirical use of antibiotics should be avoided except in cases where the patient's condition is urgent and severe, as this may complicate the final diagnosis, and S. Vanderschueren [25] et al. found in a study on the prognosis of FUO patients that empirical antibiotics were not needed for stable patients. Overuse of antibiotics often poses a threat of resistance to clinical anti-infective therapy [26], so early diagnosis and rational adjustment of antibiotics are important in the management of FUO. In this project, 24 patients (72.7%) in the experimental group adjusted or discontinued antibiotics after applying Onco-mNGS to identify the cause of the disease, and they had a good prognosis.

Thirdly, Onco-mNGS significantly shortens the time for the FUO etiological screening. The time for Onco-mNGS is 13–15 h, compared to 2–5 days [27, 28] for cultures and even longer for tumor screening. In this study, 59 patients have been confirmed the etiology. The mean time period to clarify or exclude infection/tumor in the experimental group was approximately 7.3 days shorter than that of the control group, which greatly improved the efficiency of aetiological screening for FUO.

This result showed that the use of antibiotics significantly reduced the culture detected efficiency, but has little effect on the Onco-mNGS results. Because mNGS is used to detect the nucleic acid of pathogens, the results are not affected by the previous use of antibiotics. It can be seen from the clinical data of this study that in China, most of the patients with fever to be investigated received empirical anti-infection treatment before the examination of pathogenic microorganisms, which often resulted in negative results of routine etiological examination. Therefore, mNGS has more advantages in the etiological diagnosis of such patients who have previously received empirical treatment.

Limitation exists in this study. The sample size included in this study was small, and the false-positive calls from mNGS are not subsequently verified using an orthogonal method such as target-specific polymerase chain reaction. The relatively high price of Onco-mNGS limits the wide application of this examination in clinical practice. However, for patients with FUO, it can significantly reduce the average length of stay, avoid unnecessary medical examinations and the use of antibiotics, and finally reduce the consumption of overall medical resources.

In summary, Onco-mNGS has significantly contributed to the improvement of the aetiological diagnosis of FUO in terms of sensitivity, specificity, accuracy and timeliness. We suggest patients admitted to the hospital after a Minimal FUO test fail to confirm the diagnosis immediately undergo Onco-mNGS testing to improve early diagnosis.

## Supplementary Information

Supplementary Material 1.

Supplementary Material 2.

Supplementary Material 3.

Supplementary Material 4.

Supplementary Material 5.

Supplementary Material 6.

Supplementary Material 7.

Supplementary Material 8.

Supplementary Material 9.

## Data Availability

The datasets presented in this study can be found in NCBI with the SRA accession no. PRJNA865023. The raw data of the chromosome copy variation and the picture of each patient were deposited in, or linked to, Zenodo (https://zenodo.org/record/7982217). The raw data of the chromosome copy variation and the picture of each patient were deposited in, or linked to, Zenodo (https://zenodo.org/record/7982217).
