# Supplementary figures and images for: Onco-mNGS facilitates rapid and precise identification of the etiology of fever of unknown origin: a single-centre prospective study in North China
Source: BMC Infect Dis. 2024 Dec 28;24:1475. doi: 10.1186/s12879-024-10383-3 (PMC11682622; doi:10.1186/s12879-024-10383-3)

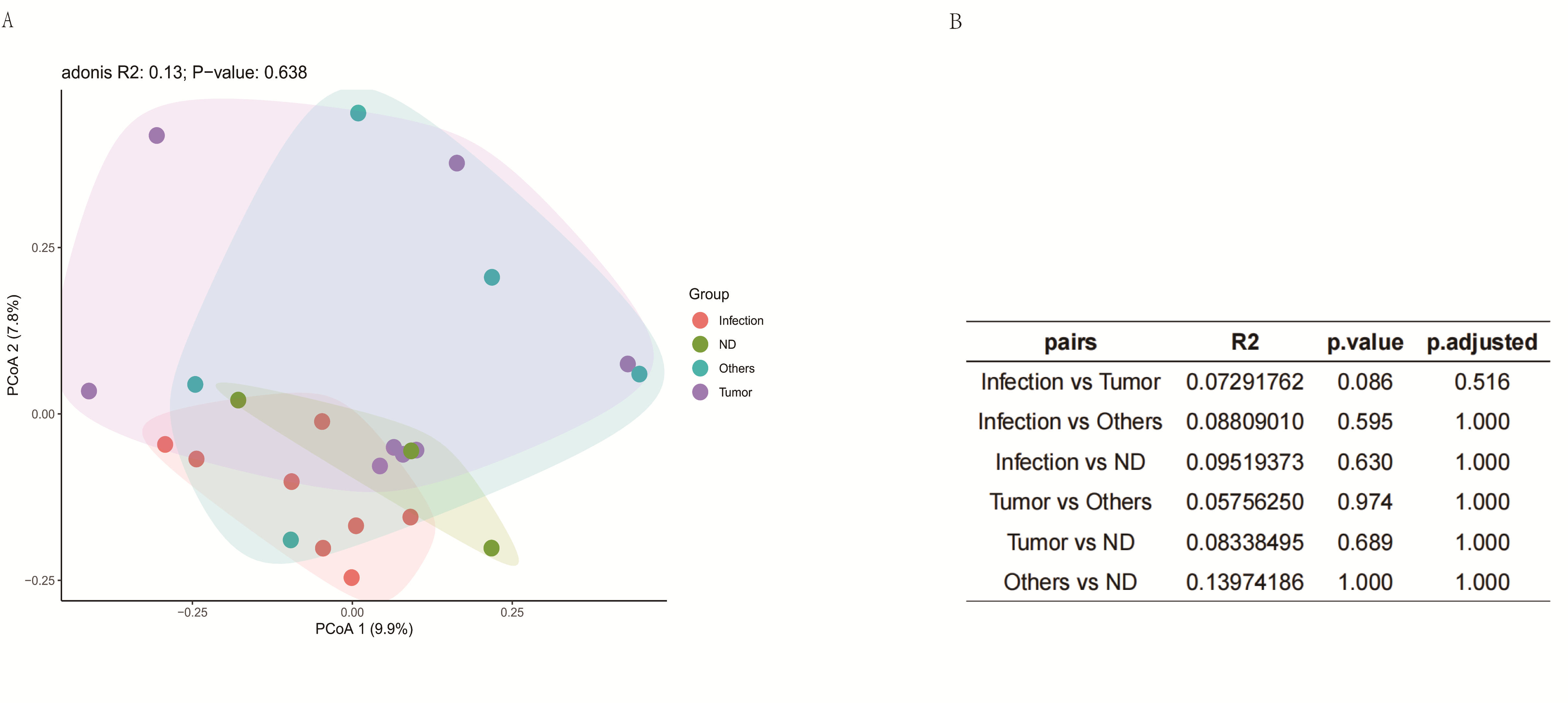

Supplement: Supplementary file 2 — Supplementary Material 2. [file 12879_2024_10383_MOESM2_ESM.tif]

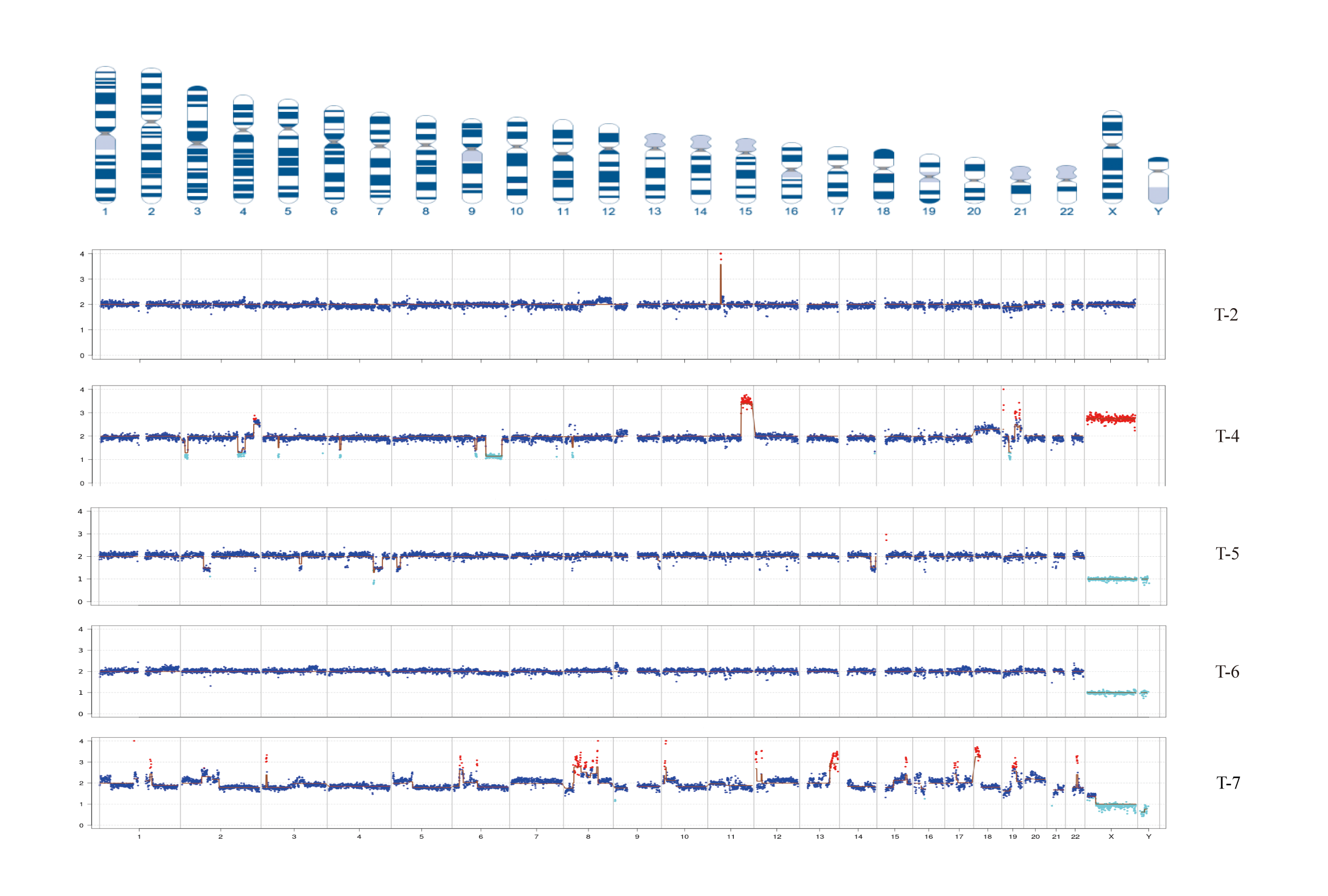

Supplement: Supplementary file 3 — Supplementary Material 3. [file 12879_2024_10383_MOESM3_ESM.tif]

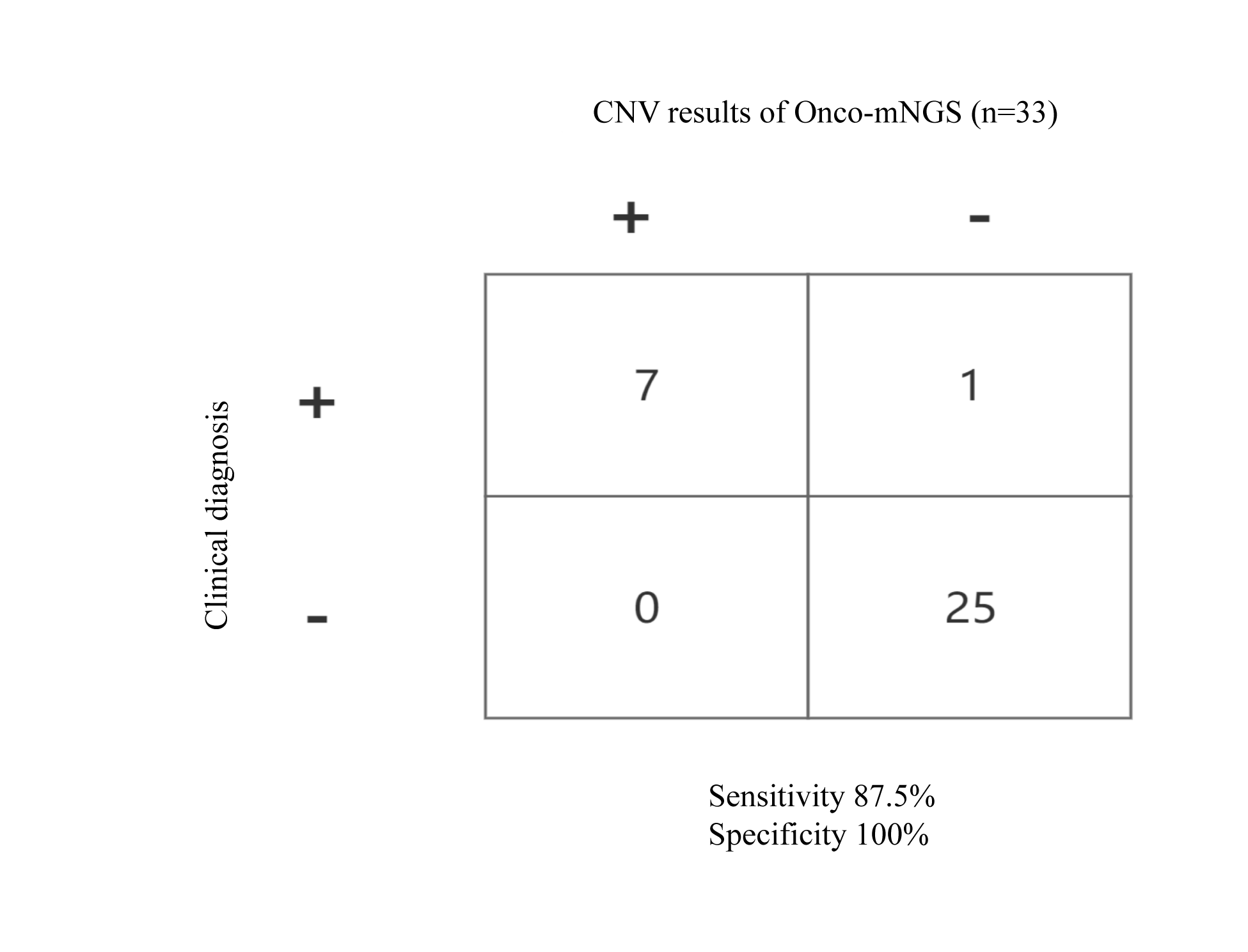

Supplement: Supplementary file 4 — Supplementary Material 4. [file 12879_2024_10383_MOESM4_ESM.tif]

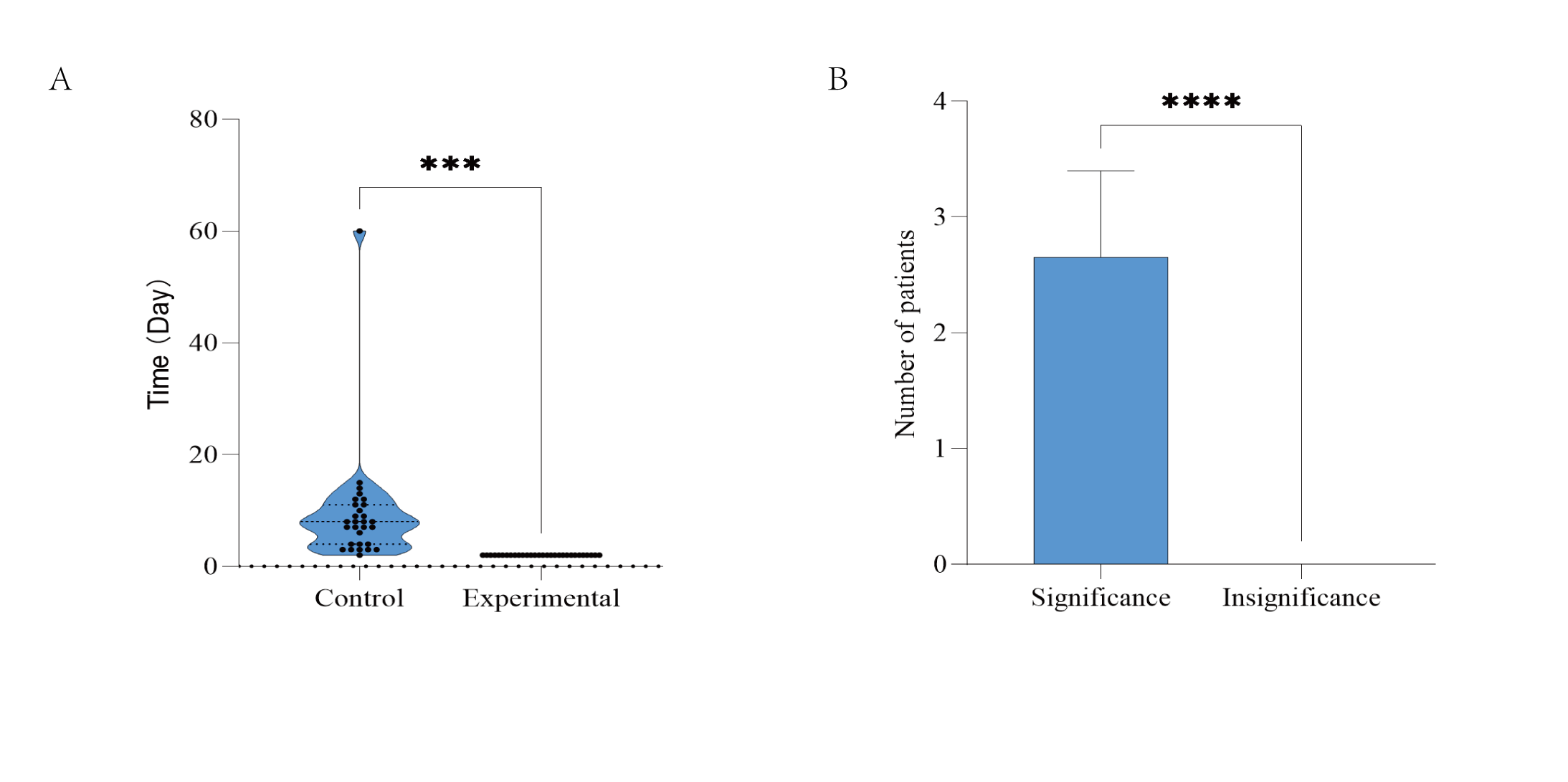

Supplement: Supplementary file 5 — Supplementary Material 5. [file 12879_2024_10383_MOESM5_ESM.tif]
