# Supplementary material for: Onco-mNGS facilitates rapid and precise identification of the etiology of fever of unknown origin: a single-centre prospective study in North China
Source: BMC Infect Dis. 2024 Dec 28;24:1475. doi: 10.1186/s12879-024-10383-3 (PMC11682622; doi:10.1186/s12879-024-10383-3)
Supplement: Supplementary file 6 — Supplementary Material 6. [file 12879_2024_10383_MOESM6_ESM.docx]

**Supplementary Figure 1 Samples composition of enrolled patients.**

**Supplementary Figure 2 Microecological Beta diversity analysis of blood samples in experimental group.** A) The greater the difference in PCoA index, the greater the evolutionary distance between the samples, B) The results showed that there was a certain trend of difference in evolutionary distance between the samples of infection and tumor patients. No significant differences had been detected.

**Supplementary Figure 3 CNV signal pattern of patients with tumor etiology of FUO.** These images show abnormal CNVs of patients (T-2, T-4, T-5, T-6, T-7).

**Supplementary Figure 4 CNV contingency table.** The clinical gold standard is histopathological examination, cytological examination or microscopic examination.

**Supplementary Figure 5 The significance of Onco-mNGS detection in clinical diagnosis and treatment.** A) The time to clinical identification or exclusion of infection/tumor was significantly shortened in the experimental and control groups, B) The treatment group combined with the clinical situation to analyze the clinical diagnosis or therapeutic significance of the results of Onco-mNGS, Significance (n=26), Insignificance (n=7), p＜0.01.
