# Supplementary material for: Onco-mNGS facilitates rapid and precise identification of the etiology of fever of unknown origin: a single-centre prospective study in North China
Source: BMC Infect Dis. 2024 Dec 28;24:1475. doi: 10.1186/s12879-024-10383-3 (PMC11682622; doi:10.1186/s12879-024-10383-3)
Supplement: Supplementary file 7 — Supplementary Material 7. [file 12879_2024_10383_MOESM7_ESM.pdf]

Table S-1 Labortary Findings of Patients.

	Experimental group（n=33）	Control group (n=32)
WBC count, 10^6 cells/μL	8.84 (1.13, 20.81)	7.51 (1.42,12.8)
＜4	5 (15.15%)	3 (9.375%)
4-10	16 (48.49%)	20(62.5%）
＞10	12 (36.36%)	9（28.125%）
NE,%	50.29 (0.373,89.2)	64.83 (27,88.3)
＜40%	11 (33.33%)	3 (9.375%)
40%-75%	14 (42.42%)	23 (71.875%)
＞75%	8 (24.24%)	6 (18.75%)
Lym,%	26.527 (0.068,88.3)	24.878 (6.5,64.8)
≤20%	20 (60.61%)	12 (37.5%)
20%-50%	7 (21.21%)	18 (56.25%)
≥50%	6 (18.18%)	2 (6.25%)
CRP, mg/L	112.23 (4.7,278.2)	78.27 (4.9,228)
＜10	1 (3.03%)	3 (9.375%)
10-50	8 (24.24%)	11 (34.375%)
51-100	7 (21.21%)	8 (25%)
＞100	17 (51.52%)	10 (31.25%)
PCT, ng/mL	2.272 (0.005,61.1)	0.216 (0.02,1.2)
＜0.1	8 (24.24%)	13 (40.625%)
0.1-0.24	10 (30.3%)	7 (21.875%)
0.25-0.5	7 (21.21%)	10 (31.25%)
＞0.5	8 (24.24%)	2 (6.255)
ESR	48.59 (4,90)a	40.56 (3,85)
＜20	4 (12.5%)	5 (15.625%)
20-50	14 (43.75%)	16 (50%)
51-90	14 (43.75%)	8 (25%)
＞90	0	0

WBC, white blood cells; CRP, C-reactive protein; PCT, procalcitonin; ESR, erythrocyte sedimentation rate

[a], This examination was performed in 32 out of 33 patients. Data were presented as n(%) or means (range).
