# Supplementary material for: Onco-mNGS facilitates rapid and precise identification of the etiology of fever of unknown origin: a single-centre prospective study in North China
Source: BMC Infect Dis. 2024 Dec 28;24:1475. doi: 10.1186/s12879-024-10383-3 (PMC11682622; doi:10.1186/s12879-024-10383-3)
Supplement: Supplementary file 8 — Supplementary Material 8. [file 12879_2024_10383_MOESM8_ESM.pdf]

Table S-2 Antibiotic therapy before definite diagnosis.

| | Experimental group (n=33) | Control group (n=32) |
|---|---|---|
| No antibiotics | 4 | 8 |
| Single anti-bacterial | 16 | 20 |
| Combined anti-bacterial | 11 | 1 |
| Combined anti-bacterial and anti-virus | 0 | 2 |
| Combined anti-bacterial and anti-fungal | 2 | 1 |
