# Supplementary material for: Onco-mNGS facilitates rapid and precise identification of the etiology of fever of unknown origin: a single-centre prospective study in North China
Source: BMC Infect Dis. 2024 Dec 28;24:1475. doi: 10.1186/s12879-024-10383-3 (PMC11682622; doi:10.1186/s12879-024-10383-3)
Supplement: Supplementary file 9 — Supplementary Material 9. [file 12879_2024_10383_MOESM9_ESM.pdf]

Table S-3 Diagnostic results of tumor cases

Sample ID	Tumor		Microorganism		Imaging and biochemical examinations
	Clinical diagnosis	CNV	mNGS	Culture	
T-1	Pleural mesothelioma	Negtive	Negtive	Negtive	CT: right pleural effusion and bilateral localized pleural thickening; Cytology exams of pleural fluid: Mesothelial dysplasia
T-2	Tumor fever	Positive	Negtive	Staphylococcus sciuri	CT: Multiplelymphadenopathyin inguinal regions of the abdominal cavity;thickening of the wall ofrectosigmoid junction.Softtissuedensityshadowintheleftureter. Endometrial biopsy: severe dysplasia of endometrial glands with local cancer, which tended to be high-grade serous carcinoma. Immunohistochemistry: CD10(-), CK(+), ER（-）, Napsin-A(+), Vimentin(-), PR(-), WT1 (-), PAX8(+), Ki-67(80%+).
T-3	Lymphoma	Positive	Negtive	Negtive	Bone marrow: B-celllymphomawithbonemarrowinfiltration
T-4	Lymphoma	Positive	CMV	Negtive	PET-CT: Diffuse hypermetabolism of the bone marrow; Bone biopsy: DLB-CL
T-5	Tumor fever	Positive	Negtive	Staphylococcus hominis	According to the physical examination and treatment, clinicians thought that fever caused by solid tumors or hematological tumors was more likely
T-6	Lymphoma	Positive	EB，CMV	Negtive	Inguinallymphnodebiopsy: Lymphatic dysplasia<br>Immunohistochemistry: CD3(+), CD68(+), CKCL-13(+), Ki-67(40%+), S-100(+), CD20(+), CD21(+), CD5(+), CD10(-), Bcl-6(-), Bcl-2(+), CD30(+), CK(-), PD-1 (-), CD4(+), CD8(-), LCA (+), CD35(-), CD163(+), CDla(-), Vimentin(+), p53(+),MPO(+)
T-7	Urinary bladder malignant tumor with multiple metastases	Positive	Negtive	Negtive	Postoperative multiple metastasis from bladder cancer
T-8	B-cell lymphoma	Positive	Negtive	Negtive	Liver biopsy: Intravascular diffuse large B-cell lymphoma<br>Immunohistochemistry: CK19（+）, CD3(+), CD34(+), HBsAg(-), HBcAg (-), IgG4(>5/50HPF), CD163(+), CD20(+), Ki-67(+), Pax-5(+), CD10(-), Bcl-6(+), CD30(-)

Abbreviations : DLB-CL , Diffuse large B cell lymphoma.
